# Intense proliferation of rDNA sites and heterochromatic bands in two distantly related *Cuscuta* species (Convolvulaceae) with very large genomes and symmetric karyotypes

**DOI:** 10.1590/1678-4685-GMB-2019-0068

**Published:** 2020-06-15

**Authors:** Amália Ibiapino, Miguel Ángel García, Mihai Costea, Saša Stefanović, Marcelo Guerra

**Affiliations:** 1 Universidade Federal de Pernambuco, Departamento de Botânica, Recife, PE, Brazil.; 2 University of Toronto Mississauga, Department of Biology, Mississauga, ON, Canada.; 3 Royal Botanic Gardens Kew, Richmond, Surrey, United Kingdom.; 4 Wilfrid Laurier University, Department of Biology, Waterloo, ON, Canada.

**Keywords:** CMA and DAPI staining, dodders, genome size, karyotype symmetry, rDNA sites

## Abstract

The genome size varies widely among angiosperms but only a few clades present huge variation at a low phylogenetic level. Among diploid species of the genus *Cuscuta* the genome size increased enormously in at least two independent lineages: in species of subgenus *Monogynella* and in at least one species (*C. indecora*) of the subgenus *Grammica*. Curiously, the independent events lead to similar karyotypes, with 2n = 30 mostly metacentric chromosomes. In this paper we compared the patterns of heterochromatic bands and rDNA sites of *C. indecora* and *C. monogyna*, aiming to evaluate the role of these repetitive fractions in these karyotypes. We found out that the large genomes of these species were incremented by a huge number of small heterochromatic CMA^+^ and DAPI^+^ bands and 5S and 35 rDNA sites, most of them clearly colocalized with CMA^+^ bands. Silver nitrate impregnation revealed that the maximum number of nucleoli per nucleus was low in both species, suggesting that some of these sites may be inactive. Noteworthy, the tandem repeats did not generate large bands or sites but rather dozens of small blocks dispersed throughout the chromosomes, apparently contributing to conserve the original karyotype symmetry.

## Introduction

The genus *Cuscuta* L. (Convolvulaceae), commonly known as dodders, consists of approximately 200 species (Yuncker, 1932; Costea *et al.*, 2015) of hemiparasitic or holoparasitic herbs, and is nearly cosmopolitan in distribution. Taxonomically, this group is difficult due to interspecific hybridizations, infraspecific variability, and strong vegetative reduction associated with diminished or complete lack of photosynthetic activity resulting in morphological parallelism among species (Stefanović and Costea, 2008; Costea *et al.*, 2015). Cytologically, however, *Cuscuta* is one of the plant genera with the largest variability in genome size (1C = 0.48 pg to 1C = 32.77 pg) and perhaps the only plant genus with species having both monocentric and holokinetic chromosomes (Pazy and Plitmann, 1995; McNeal *et al.*, 2007; Leitch *et al.*, 2010; Guerra *et al.*, 2019).

*Cuscuta* is currently divided into four subgenera: *Monogynella*, represented by approximately 15 species of the Old World; *Grammica*, comprising about 150 species mostly from the Americas; *Cuscuta*, with 20-25 species originally from the Old World; and *Pachystigma*, a small group of five species endemic to South Africa (Costea *et al.*, 2015). The scarce chromosome counts available for only 35 species of this genus indicate that most diploid species present 2n = 28 or 2n = 30, and that each subgenus followed a distinct karyotype trend. There are very large chromosomes in *Monogynella* (Pazy and Plitmann, 1995), holokinetic chromosomes in the subgenus *Cuscuta* (García and Castroviejo, 2003), large DNA content variation (2C = 0.96 to 65.54 pg) in *Grammica* (McNeal *et al.*, 2007; Kubesová *et. al.*, 2010), and strongly bimodal karyotypes in *Pachystigma* (García *et al.*, 2019). Phylogenetically, the genus *Cuscuta* is clearly nested within Convolvulaceae (Stefanović *et al.*, 2002; Stefanović and Olmstead, 2004), a family otherwise characterized by small genome size (2C ≤ 4.5 pg) (Kew Plant DNA C-values Database; http://data.kew.org/cvalues/), with predominantly symmetrical karyotypes, small chromosomes, and chromosome number 2n = 30 in most genera and species (e.g., Yen *et al.*, 1992; Pitrez *et al.*, 2008).

**Figure 1:**
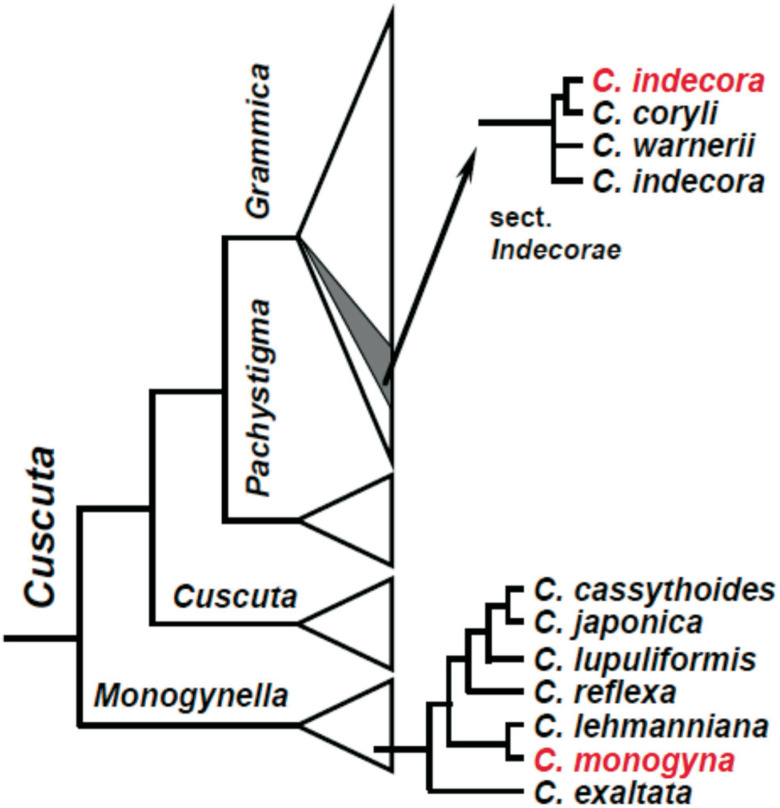
Schematic overview of the phylogenetic relationships in *Cuscuta* derived from sequence data from plastid (*trnL-F*, *rbcL*) and nuclear (nrITS, nrLSU) sources and analyzed with a range of analytical methods (modified from Costea *et al.*, 2015). Infrageneric classification is provided above branches on the left, and more detailed relationships among species of *Cuscuta* sect. *Indecorae* and *Cuscuta* subgenus *Monogynella* are shown on the right (Stefanović *et al.*, 2007; García *et al.*, 2014). Species of particular interest for this study are highlighted in red. Note that *C. indecora* is not monophyletic as currently circumscribed.

Extreme increase in genome size may have occurred at least twice during the evolution of the genus *Cuscuta*: across species of the subgenus *Monogynella* (*C. exaltata* Engelm., 2C = 41.86 pg; *C. lupuliformis* Krock., 2C = 44.93 pg), and in at least one species of the subgenus *Grammica* [*C. indecora* Choisy, with 2C = 65.54 pg (McNeal *et al.*, 2007)], placed in the small section *Indecorae*. Likewise, the largest chromosome size for the genus was reported for two other *Monogynella* species, *C. reflexa* Roxb. (Kaul and Bahn, 1974) and *C. monogyna* Vahl. (Pazy and Plitmann, 1995; García and Castroviejo, 2003), and for *C. indecora* (Fogelberg, 1938; García *et al.*, 2019). Figure 1 illustrates the phylogenetic relationships among *Cuscuta* subgenera and the relative position of *C. indecora* and *C. monogyna* (based on García *et al.*, 2014; Stefanović *et al.*, 2007; Costea *et al.*, 2015). Note that *C. indecora* is not monophyletic as currently circumscribed.

In spite of the huge variation in chromosome size across the genus, all *Monogynella* and *Grammica* species display symmetrical or nearly symmetrical karyotypes (García *et al.*, 2019), suggesting that the evolution of the biggest genomes occurred without structural rearrangements that could change the chromosome morphology. Large genome expansions, as observed in *Cuscuta*, are more commonly due to a burst of one or a few retroelements and/or satellite DNA sequences (Michael, 2014; Garrido-Ramos, 2017). Because satellite DNA families are organized in blocks of tandemly repeated sequences, they are usually not included in the genome sequencing and hence its real role in the genome size variation is poorly known. Recent analyses of satellite DNA families detected by next-generation sequencing (NGS) combined with appropriate bioinformatic tools and chromosome *in situ* hybridization (FISH) revealed that most satellites colocalized with the classical chromosome bands (Ruiz-Ruano *et al.*, 2016; Palacios-Gimenez *et al.*, 2017; Robledillo *et al.*, 2018).

Chromosome banding using base-specific fluorochromes, mainly the DNA ligand chromomycin A3 (CMA) and 4’,6-diamidino-2-phenylindole (DAPI), which bind preferentially to AT-rich and GC-rich sequences respectively, reveal most of the heterochromatin content of the karyotype (Barros e Silva and Guerra, 2010). The only *Cuscuta* species investigated by chromosome banding and FISH using 5S and 35S rDNA probes were *C. approximata* Bab. of subgenus *Cuscuta* (Guerra and García, 2004), and the three species of subgenus *Grammica* section *Denticulatae* (Ibiapino *et al.*, 2019), which exhibited a variable number of CMA and DAPI bands and rDNA sites.

We undertook the current work to estimate the genome size of *C. monogyna* and *C. indecora* and to provide a detailed karyotype analysis of both species, including the heterochromatic bands and rDNA sites, with an ultimate aim to evaluate the role of the repetitive fractions in these convergent genome expansions and maintenance of karyotype symmetry in these independent lineages.

## Material and Methods

### Plant material

One sample of *Cuscuta monogyna* and four samples of *C. indecora* were analyzed. The samples investigated with their collection information, voucher number, herbaria where the vouchers are deposited and karyotype data are presented in Table 1. Seeds of both species were scarified with concentrated sulfuric acid for 60-90 s, rinsed several times with distilled water, and germinated on wet filter paper in Petri dishes. Seedlings were cultivated in the greenhouse of the University of Toronto Mississauga, using coleus [*Plectranthus scutellarioides* (L.) R. Br.] as a host. Seedlings of *C. monogyna* were also cultivated in the Federal University of Pernambuco (Recife, Brazil), where most cytological analyses and genome size estimation were conducted. Vouchers are deposited in the herbarium of the University of Toronto Mississauga (TRTE).

**Table 1:** Samples of *Cuscuta monogyna* and *C. indecora* investigated, with respective voucher, collection locality, chromosome number observed in meiosis (n) or mitosis (2n), and genome size (2C).

Species	Voucher	Locality	*n*	2*n*	2C ± CV
*Cuscuta monogyna* Vahl	UTM-1348	Israel: Kursi; dat: 2012	15	30	66.08 ± 0.27
*C. indecora* Choisy	UTM-1568	Supplied by SAGARPA (Secretaría de Agricultura, Ganaderia, Desarrollo Rural, Pesca y Alimentación), México	15	30	45.58 2.66
	Stefanović SS-16-74, TRTE	USA: New Mexico; Chaves Co., Roswell, corner E McCune and S Main St (Hwy 285); dat: 16 Aug 2016	15		
	Stefanović SS-16-53, TRTE	USA: New Mexico; Socorro Co., on Pueblitos Rd., 1/3 mi E of Escondida Bridge Park (1/2 mi E of Hwy 408); dat: 9 Aug 2016	15	30	
	Stefanović SS-16-77 b, TRTE	USA: New Mexico; Chaves Co., Bottomless Lakes Rd., 3 mi S of Hwy 380. 17 August 2016. 33 21’31″N 104 20’16″W			50.03 ± 0.05

### Slide preparation and chromosome staining

For mitotic analyses, shoot tips were pretreated in 0.2% colchicine for 24 hours at 10 °C, fixed in a 3:1 ethanol-acetic acid solution, and subsequently stored at −20 °C. For meiotic analyses, young flower buds were directly fixed and stored as above. For cytological analyses, we followed the same protocols used for other *Cuscuta* species (Guerra and García, 2004). The fixed material was washed in distilled water, digested in a 2% (w/v) cellulase (Onozuka)/20% (v/v) pectinase (Sigma) solution at 37° C for 60 min, squashed in a drop of 45% acetic acid and the coverslip removed in liquid nitrogen.

For CMA/DAPI staining, the slides were aged for three days, and stained for 60 min with CMA (0.1 mg/mL) and 30 min with DAPI (1 μg/mL). The slides were then maintained in the dark for three days before analysis under an epifluorescence Leica DMLB microscope. The images were captured with a Cohu CCD video camera using Leica QFISH software and were later optimized for better contrast and brightness using Adobe Photoshop CS3 version 10.0.

*In situ* hybridization was performed according to Pedrosa *et al.* (2002), with small modifications. A 500 bp 5S rDNA clone (D2) of *Lotus japonicus* (Regel) K. Larsen, labelled with Cy3-dUTP (Amersham), and a 6.5 kb 35S rDNA clone (R2) of *Arabidopsis thaliana* (L.) Heynh., labelled with digoxigenin-11-dUTP, were used as probes. The labelling was done by nick translation. The 35S rDNA probe was detected with sheep anti-digoxigenin FITC (Roche) and amplified with rabbit anti-sheep FITC (Dako). The hybridization mix contained formamide 50% (v/v), dextran sulphate 10% (w/v), 2 SSC and 5 ng/μL of each probe. Both chromosomes and probes were denatured at 75 °C for 10 min and hybridized at 37 °C for 18 h. The post-hybridization washes were performed in 0.1 SSC at 42 °C for 15 min, the slides were counterstained with DAPI 2 μg/mL and mounted in Vectashield H-1000 (Vector). The cells previously acquired with CMA/DAPI staining were photographed again and the images were optimized as before.

Because both species presented a high number of rDNA sites, we analyzed the number of nucleoli per nucleus by silver nitrate impregnation to check if there was a real increment in the number of active nucleolus organizer regions (NORs). In this case, a drop of 50% silver nitrate diluted in distilled water was added to slides containing a large number of interphase nuclei from young shoot tips, covered with a coverslip, and maintained at 60 °C in water bath for 1-2 hours [slightly modified from Kodama *et al.* (1980)]. When nucleoli were clearly differentiated, the slides were washed, air dried, and mounted in glycerol.

### Chromosome length measurement and flow cytometry

Chromosome size estimation was based on measurements of the four best metaphases of each species, using Adobe Photoshop CS3 software version 10.0. Chromosome arm ratio (length of the long arm/length of the short arm) was used to classify chromosomes as metacentric (1.00–1.49) or submetacentric (1.50–2.99), according to Guerra (1986). For flow cytometry, a suspension of nuclei from shoot tips was prepared using WPB buffer (Loureiro *et al.*, 2007). The cells were stained with propidium iodide and the nuclear DNA amount was estimated using a CyFlow SL flow cytometer (Partec, Görlitz, Germany). As an internal control young leaves of *Vicia faba* L. ssp. *faba* ‘Inovec’ (2C = 26.9 pg; Doleel *et al.*, 1991) were used. The final 2C value was based on three different measurements for each sample using the equation “Sample peak mean/Standard peak mean 2C DNA content of internal control (pg)” and the software FloMax (Partec) for data processing.

## Results

### Chromosome number, size, morphology and DNA amount

**Figure 2:**
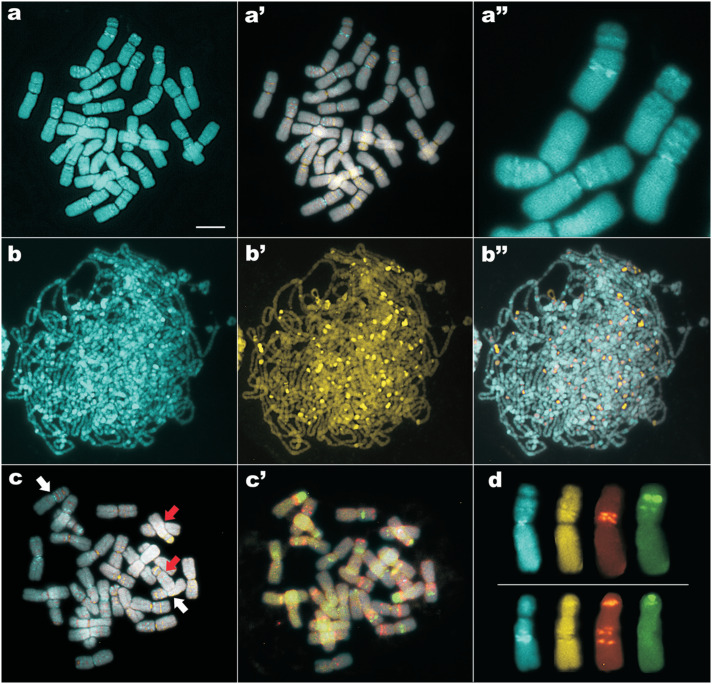
CMA/DAPI bands and rDNA sites in *Cuscuta monogyna*. (a-a❞) Metaphase showing DAPI bands (a), DAPI and CMA merged images (a❜) and enlarged images of some chromosomes with several DAPI bands (a❞). (b-b❞) Zygotene stained with DAPI (b), CMA (b❜) and merged images (b❞). (c, c❜) Metaphase showing CMA and DAPI bands (c) and rDNA sites (c❜). White and red arrows indicate the chromosome pairs bearing, respectively, the first and the second largest pairs of 35S rDNA sites. (d) First (upper row) and second (lower row) largest pairs of satellited chromosomes from another metaphase showing heterochromatic bands and rDNA sites. Observe that the centromere in the metacentric pair was DAPI^+^/CMA^−^ whereas in the other pair it was negative for DAPI and undifferentiated for CMA. Blue = DAPI; yellow = CMA; orange = 5S rDNA; green = 35S rDNA. Bar in (a) corresponds to 10 μm (not valid for a❞ and d).

**Figure 3:**
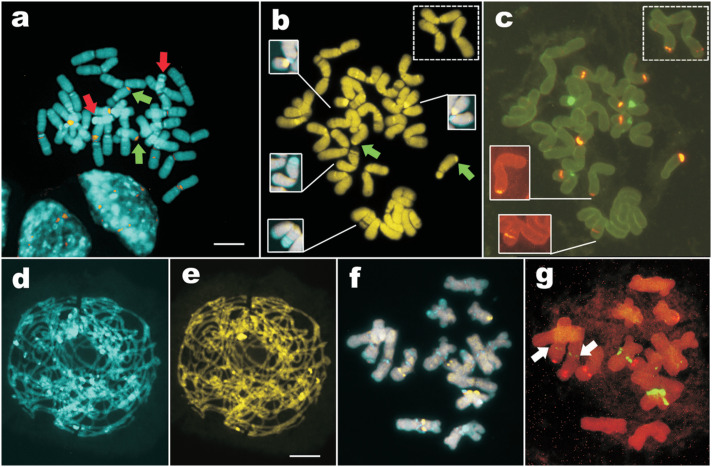
CMA/DAPI bands and rDNA sites in *Cuscuta indecora*. (a) Metaphase showing merged DAPI and CMA images (red arrows = terminal DAPI bands; green arrows = terminal CMA bands). (b, c) Metaphase with CMA bands (b) and 5S (orange) and 35S (green) rDNA sites (c). Three chromosomes were outside the picture (dashed square). Insets in (b) show chromosome pair with proximal secondary constriction (up) and terminal DAPI bands (down) and in (c) show weak sites. Green arrows in (a) and (b) point to CMA^+^ bands co-localized with 5S rDNA sites. (d, e) Zygotene stained with DAPI (d) and CMA (e). (f, g) Diakinesis showing main bands (f) and rDNA sites (g) with two weak 35S rDNA sites (white arrows). Bar in (a) corresponds to 10 μm.

The two species displayed 2n = 30 large chromosomes with similar symmetrical karyotypes (Figures. 2 and 3). Secondary constrictions were observed on a single pair of metacentric chromosomes in both species, although they were not always visible. They were located interstitially in *C. monogyna* (Figure 2d) and proximally in *C. indecora* (upper insets in Figure 3b). In meiosis, both species presented regular chromosome pairing with 15 bivalents. In *C. monogyna*, there were 13 metacentric pairs varying from 14.49 to 21.60 μm (arm ratio: 1.05 to 1.29) and two submetacentrics displaying 12.41 and 13.65 μm in length (arm ratio: 1.91 and 2.59). *Cuscuta indecora* (UTM-1568) had 14 metacentric pairs varying in size from 13.66 to 18.25 μm (arm ratio:1.00 to 1.31) and one submetacentric with an average size of 10.77 μm (arm ration: 2.50). The genome size was higher in *C. monogyna* (2C = 67.58 ± 0.27 pg) than in *C. indecora.* The two samples of *C. indecora* analyzed by flow cytometry presented different results: 2C = 50.03 ± 0.05 pg (SS-16-77b) and 2C = 45.58 ± 2.66 pg (UTM-1568). The former estimation was obtained from shoot tips of young plantlets whereas the latter one was from an adult plant growing in greenhouse. However, this variation may also be due to differences between populations as indicate in Figure 1.

### CMA/DAPI bands and rDNA sites

*Cuscuta monogyna* showed a very large number of small CMA^+^/DAPI^−^ and CMA^−^/DAPI^+^ bands, resulting in a stripped appearance of some chromosome arms (Figure 2a-a❞). Most of the bands were weakly contrasted, especially the CMA^+^ ones, and the vast majority were located on interstitial positions, although there were also some terminal, proximal, and a few centromeric bands. The whole heterochromatin of *C. monogyna* was more clearly seen in early-pachytene nuclei (Figure 2b-b❞), allowing to count almost 90 CMA^+^/DAPI^−^ bands and near 80 DAPI^+^/CMA^−^ bands. The exact number of bands and rDNA sites was difficult to ascertain because some of them were too closely positioned, too small, or weakly labelled.

*In situ* hybridization revealed nearly 36 sites of 5S rDNA and 30 sites of 35S rDNA in *C. monogyna* (Figure 2c). Most sites were interstitials, except three pairs of 5S and one pair of 35S rDNA, which were terminally located. Noteworthy, only six pairs of 35S rDNA sites and near half of the 5S rDNA sites of *C. monogyna* were clearly colocalized with CMA^+^ bands (Figure 2c, c❜). Although several rDNA sites were located very close to DAPI^+^ bands, detailed analysis revealed that none of them were colocalized with DAPI^+^ bands.

Each chromosome pair of *C. monogyna* had at least one or more heterochromatic bands and rDNA sites, allowing for an easy identification of every chromosome pair. The two pairs bearing the largest 35S rDNA sites of the complement illustrated very well the use of these markers for chromosome identification. The largest 35S rDNA site was located on the shorter arm of a metacentric pair, colocalized with a weak CMA^+^ band negatively stained by DAPI (white arrows in Figure 2c and selected chromosomes from another metaphase in Figure 2d upper row). This chromosome arm also had a smaller 35S rDNA site and two 5S rDNA sites. The second largest 35S rDNA site was located on the short arm of a submetacentric pair, adjacent to a 5S rDNA site (red arrows in Figure 2c and lower row in Figure. 2d). The long arm of this chromosome exhibited two other 5S rDNA and the largest DAPI^+^ band of the complement. Observe that the centromere in the metacentric pair was DAPI^+^/CMA^−^ whereas in the other pair it was negative for DAPI and undifferentiated for CMA. The 5S rDNA sites on both chromosome pairs were positively differentiated by CMA.

In *C. indecora*, the number of CMA and DAPI bands was smaller than in *C. monogyna*. Large DAPI^+^ bands were only observed in the terminal region of a single chromosome pair and in the proximal region of another pair (red arrows in Figure 3a and insets in Figure 3b). Additionally, there was a single proximal band in most chromosomes and several weakly differentiated interstitial and terminal DAPI^+^ bands (Figure 3a). The largest CMA^+^ band was located on the proximal region of a metacentric pair (upper insets in Figure 3b) and several fine interstitial or terminal CMA^+^ bands were observed (Figure 3b). Early-pachytene cells showed a much smaller number of heterochromatic bands in *C. indecora* when compared with *C. monogyna*, with a predominance of DAPI^+^ bands (Figure 3d, e).

Concerning rDNA sites, *C. indecora* exhibited five pairs of 5S rDNA sites, all of which were colocalized with CMA^+^ bands, which were sometimes poorly differentiated (Figure 3b, c, and 3f, g). The largest 5S rDNA site was located on the long arm termini of the only submetacentric pair, co-localized with a CMA^+^ band (green arrows in Figure 3a, b). There were only two pairs of proximal 35S rDNA sites (Figure 3c), the largest of which was colocalized with the largest CMA^+^ band (Figure 3b, c), and sometimes distended as a secondary constriction. One or two pairs of small 35S rDNA sites were sometimes observed (white arrows in Figure 3g). Although all rDNA sites appear to colocalize with CMA bands, some CMA^+^ bands did not colocalized with none of the rDNA sites (compare Figure 3b, c, and 3f, g.).

**Fig. 4:**
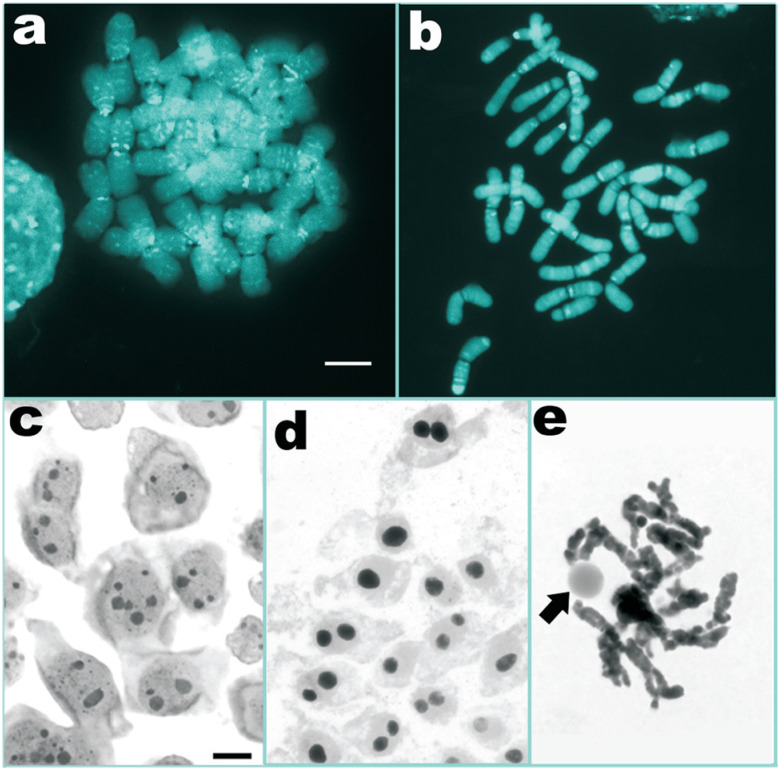
Heterochromatic bands observed after FISH (a, b) and nucleoli (c-e) in *C. monogyna* (a, c) and *C. indecora* (b, d, e). Arrow in (e) points to the single nucleolus of a diplotene. Bar in (a) and (c) corresponds to 10 μm.

After FISH, DAPI stained chromosomes of both species revealed numerous small bands and a few relatively large ones (Figure 4a, b). Most of these bands corresponded to the DAPI bands observed in the direct CMA/DAPI staining, which were now best contrasted. In *C. monogyna* they were observed as very fine interstitial dot-like bands and a few proximal larger ones (Figure 4a), whereas in *C. indecora* there were proximal bands in most chromosomes, a few terminal ones and several weak interstitial bands (Figure 4b). The number of DAPI-FISH bands per chromosome arm varied from 0 to 6 in *C. monogyna* and from 0 to 5 in *C. indecora*.

In order to evaluate if the proliferation of 35S rDNA sites resulted in a proportionally large number of nucleoli, we analyzed 3,052 nuclei of *C. monogyna* and 1,225 nuclei of *C. indecora* by silver staining. In *C. monogyna*, the number of nucleoli varied from one to six, with most nuclei displaying two large nucleoli and a few smaller ones (Figure 4c). The number of nucleoli per nucleus in most cells of *C. indecora* was only one or two (Figure 4d), while a very few cells had three or four nucleoli. In all pachytene cells of *C. indecora* only one nucleolus was found (Figure 4e).

## Discussion

### Genome size variation

Our results confirmed that both species possess large DNA content, although the genome size estimated for *C. indecora* (2C = 45.58 and 50.03 pg) was much lower than that reported by McNeal *et al.* (2007) (2C = 65.54 pg). The different 2C values observed in the three measured samples for *C. indecora* is too high to be attributed to intraspecific variation (Greilhuber, 2005). The chromosome number of the sample quantified by McNeal *et al.* (2007) was not determined, but all other previous chromosome counts for *C. indecora* (Fogelberg, 1938; Raven *et al.*, 1965; Pinkava *et al.*, 1974), including the present three samples exhibited 2*n* = 30. The mitotic chromosome size of our sample (between 10.4 to 18.7 μm) was similar to that described by Fogelberg (1938), who found the largest chromosomes had 16-17 μm. Morphologically, *C. indecora* is a variable species, with three varieties, sharing several important floral characters with other closely related species (Costea *et al.*, 2006). Albeit limited, the available molecular evidence (García *et al.*, 2014; Stefanović *et al.*, 2007) suggests that this species is not monophyletic as currently circumscribed (Figure 1). It contains at least two distinct segregates, one of which is more closely related to members of *C. coryli* Engelm. than to other individuals of *C. indecora*. This phylogenetic distinction among populations of *C. indecora* is consistent with the diversity of genome size measurements reported here and previously (McNeal *et al.*, 2007). *Cuscuta coryli*, one of the two other species of section *Indecorae*, has 2n = 30 medium sized (4 to 8 μm) chromosomes (Fogelberg, 1938), suggesting a 2C value much smaller than in *C. indecora* and an intense genome size variation inside the section.

The genome size of *C. monogyna* (2C = 67.58 pg) is the largest one registered for *Cuscuta* species. The large genome size of *C. monogyna* and *C. indecora* is mirrored by the large size of their pollen grains; the former species exhibits the largest pollen grains in the genus (Welsh *et al.*, 2010). Actually, *C. monogyna* has one of the largest genomes reported for eudicots, being surpassed only by some species of Viscaceae and Loranthaceae (Leitch and Leitch, 2013). The 2C values of 1.7 to 2.4 pg (Ozias-Akins and Jarret, 1994; Bennett and Leitch, 2011) reported for other diploid species of Convolvulaceae with 2n = 30 or nearly 30, are at least 17 times lower than those of *C. monogyna*, leading to the hypothesis that large bursts of genome expansion occurred only in the genus *Cuscuta*. Although the exact phylogenetic position of *Cuscuta* within Convolvulaceae is still unknown (Stefanović and Olmstead, 2004), *Monogynella* shares some plesiomorphic features with nonparasitic Convolvulaceae relatives, such as the presence of xylem absent in the remaining subgenera, and some floral, fruit, and anatomical characters (García *et al.*, 2014; Wright *et al.*, 2011).

Unlike those from the subgenus *Monogynella*, the genome sizes known for the subgenus *Grammica* are at least three times smaller than that of *C. indecora* (McNeal *et al.*, 2007)*.* Similar up-and-down variation of genome size has been observed in some other plant taxa (Vallès *et al.*, 2013; Pellicer *et al.*, 2018), but rarely on such a large scale and at such a low phylogenetic level, within a relatively small genus. A similar example is found in the genus *Oxalis* (Oxalidaceae), with two peaks of high 2C values: one in the subgenus *Oxalis* (range: 0.58 to 14.59 pg) and another in the subgenus *Thamnoxys* (range:1.76 to 41.88 pg), with a 72-fold total variation (Vaio *et al.*, 2018). It is also noteworthy that the two largest genome expansions observed in *Cuscuta* species resulted in almost identical symmetric karyotypes, while in *Oxalis* they were quite distinct and asymmetrical.

### Karyotype symmetry

Assuming that the large genome expansion events were mainly due to amplification of mobile elements (El Baidouri and Panaud, 2013; Garrido-Ramos, 2017), the karyotype symmetry would: a) increase, if insertions of the new elements were equally distributed in the chromosome arms; b) decrease, if the new insertions were preferentially accumulated in some chromosome arms (Levin, 2002; Peruzzi *et al.*, 2009). Given that all *Cuscuta* subg. *Grammica* species cytologically known (García and Castroviejo, 2003; García *et al.*, 2019), as well as the non-*Cuscuta* Convolvulaceae species (Pitrez *et al.*, 2008 and references therein), display small chromosomes and symmetrical to moderately symmetrical karyotypes, we conclude that the two genomes expansions in *Cuscuta* occurred mainly by proliferation of repetitive elements which were distributed evenly along the length of the chromosome arms.

### Heterochromatin and rDNA sites

Repetitive DNA families represent over 70% of plant genomes (Michael, 2014), but for these two *Cuscuta* species, the high number of heterochromatic bands and rDNA sites have contributed greatly to the increasing of these genomes, in comparison to the other species of this group. The elevated number of 5S and 35S rDNA sites observed in *C. indecora* (14 sites) and *C. monogyna* (ca. 66 sites) seems to confirm the correlation between genome size and number of rDNA sites (Prokopowich *et al.*, 2003; Vallès *et al.*, 2013). However, *C. nevadensis,* with 2n = 30 and much smaller chromosomes, had 16 rDNA sites (Ibiapino *et al.*, 2019); therefore, this relationship is not clear for *Cuscuta* species.

Several CMA^+^ bands were colocalized with rDNA sites but the number of CMA^+^ bands in both *Cuscuta* species was higher than the number of rDNA sites, indicating that this kind of heterochromatin should be composed by at least three different types of repetitive sequences (5S rDNA, 35S rDNA, and at least a GC-rich satellite DNA sequence corresponding to the CMA^+^ bands which did not colocalize with rDNA sites). In general, 35S rDNA sites are positively stained with CMA due to the high GC content of their internal transcribed spacers (ITS) (Baldwin *et al.*, 1995) whereas the non-transcribed spacers (NTS) of 5S rDNA sites are more variable in GC content (Waminal *et al.*, 2014), and less often CMA^+^ (e.g., Cabral *et al.*, 2006). In *C. indecora* and *C. monogyna* not all 5S and 35S rDNA sites were clearly differentiated with CMA, either because the sites were too small or because they presented a variable GC content. In three other species of *Cuscuta* subgenus *Grammica* investigated with sequential CMA/DAPI and FISH staining (Ibiapino *et al.*, 2019), only the 35S rDNA sites were CMA^+^, indicating a less variable composition of their rDNA repeats.

After the FISH procedure, all DAPI^+^ bands observed by CMA/DAPI staining became better contrasted and some other bands not detected before became visible, mainly in *C. indecora*, indicating that part of the heterochromatin was neither particularly rich in GC (CMA^+^ bands) nor in AT (DAPI^+^ bands) (Barros e Silva and Guerra, 2010). Altogether, the number of heterochromatic bands in *C. monogyna* and *C. indecora* seemed to represent a significant fraction of these large genomes. However, it was not possible to estimate the proportion of heterochromatin in these karyotypes, because most bands were too small and poorly contrasted to allow a reliable measurement. Recent analyses of the plant “satellitome” by NGS and FISH, revealed a surprising diversity of satellite DNA sites (González *et al.*, 2017; Wang *et al.*, 2017; Robledillo *et al.*, 2018), suggesting that the total amount of heterochromatin in these two species may be still higher than observed by banding methods.

In spite of the much higher number of 35S rDNA sites in *C. monogyna* than in *C. indecora*, the expression of these sites, as estimated by the maximum number of nucleoli per nucleus, was relatively small and similar in both species, possibly because some of them were permanently inactivated, as in *Arabidopsis thaliana* (Chandrasekhara *et al.*, 2016), or temporarily inactive, as observed in other species (Grabiele *et al.*, 2018; Báez *et al.*, 2020). Thus, the exceeding number of rDNA sites is most likely an accidental consequence of the genome expansion rather than a selective advantage fixed during the evolution of these species.

Beside the difference in number of heterochromatic bands and rDNA sites, *C. monogyna* and *C. indecora* presented different distribution patterns of these markers. They were predominantly located on the proximal or terminal chromosome regions in *C. indecora* and randomly distributed in *C. monogyna,* suggesting that different mechanisms of rDNA site dispersion were involved. An equilocal distribution of tandem repeats, either terminal or proximal, could be promoted by non-homologous recombination between telomeric or pericentromeric regions of different chromosome pairs (Schweizer and Loidl, 1987; Pedrosa-Harand *et al.*, 2006) during the bouquet formation or Rabl orientation, an aleatory distribution of repeat arrays is most probably mediated by mobile elements (Dubcovsky and Dvorák, 1995; Raskina *et al.*, 2008; Bueno *et al.*, 2016).

## Conclusions

The huge genome expansion that occurred in two independent *Cuscuta* lineages included intensive amplification of tandemly repeated sequences without important changes in the karyotype symmetry. Our results indicate that the tandem repeats did not generate large blocks of heterochromatin but rather dozens of small heterochromatic blocks. However, the dispersed fine blocks were not enough to change the original karyotype symmetry of these species. Despite the exceptionally high number of rDNA sites, the maximum number of nucleoli per nucleus observed was relatively low, suggesting that many of these sites were permanently or temporarily inactivated. Further analyses of methylation pattern and more specific transcription experiments are necessary to demonstrate the functionality and the faith of these sites.
